# Valorization of *Ligusticum chuanxiong* hydrosol via *Eurotium cristatum* biotransformation: mechanistic insights into flavor remodeling and functional enhancement

**DOI:** 10.3389/fnut.2026.1852271

**Published:** 2026-06-12

**Authors:** Shuo Zhang, Zixuan Su, Zhuliang Duan, Lening Chen, Peng Zhang, Zhidi Li, Lin Ye, Xiaobo Yang, Yuyuan Chen, Shaojie Zeng, Yifei Wang

**Affiliations:** 1 Faculty of Flavour Fragrance and Cosmetics, Shanghai Institute of Technology, Shanghai, China; 2 Boton (Shanghai) Biotechnology Co., Ltd., Shanghai, China

**Keywords:** biofunctional property, biotransformation, *Eurotium cristatum*, flavor remodeling, *Ligusticum chuanxiong* hydrosol

## Abstract

*Ligusticum chuanxiong* hydrosol, a low-value distillation by-product, is usually discarded because of its offensive medicinal odor and pronounced bitterness. To overcome these sensory defects, this study employed *Eurotium cristatum*, a “golden flower” fungus renowned for flavor remodeling in traditional fermentation, to valorize this underexplored aqueous matrix. This represents the first systematic application of *E. cristatum* for hydrosol biotransformation. Under optimized conditions (39°C, pH 6.7, 7% inoculum), total phenolics reached 71.25 mg/L. Electronic nose and tongue analyses indicated changes in the aroma profile and a reduction in bitterness intensity from 8.0 to 4.0. GC-MS and GC-O revealed flavor remodeling through the elimination of 2,4-dimethylphenol and enrichment of pleasant aroma compounds, including α-terpineol, D-limonene, and citral. Fermentation also enhanced hypoglycemic potential, with α-amylase and α-glucosidase inhibition reaching 80 and 79%, respectively. In addition, the fermented hydrosol showed increased radical-scavenging activity (83% DPPH and 84% ABTS) and improved cellular protection by reducing intracellular ROS, increasing superoxide dismutase activity, and, at 1% inclusion, attenuating IL-6, IL-1α, and TNF-α. Overall, *E. cristatum* effectively upcycled botanical by-products into high-value functional ingredients, offering a sustainable, green strategy for simultaneously improving the sensory quality and nutritional value of plant-based foods.

## 1 Introduction

Hydrosols (hydrolates), the aqueous side-streams generated during essential oil distillation, have a long history of utilization as fragrances and traditional remedies in China (1). While currently treated as low-value distillation waste, they retain significant water-soluble precursors and bioactive trace volatiles, such as terpenes and oxygenated derivatives, positioning them as promising candidates for valorization in the food industry (2). Specifically, *Ligusticum chuanxiong* is a traditional medicinal and edible plant widely used in China, particularly in prescriptions related to promoting blood circulation, relieving headaches, and managing menstrual symptoms (3). In modern applications, it is also processed to obtain essential oil, as its volatile fraction is regarded as a key bioactive component and has been explored for use in medicinal, nutraceutical, food, and cosmetic products. During this process, substantial volumes of hydrosol are generated as an aqueous side stream (4). However, the direct application of *L. chuanxiong* hydrosol is severely hindered by profound sensory defects, notably a pungent medicinal odor, intense bitterness, and astringency (3). Furthermore, many functional phenolic and terpenoid constituents within this raw aqueous matrix exist in glycosidically bound or conjugated forms, thereby restricting their bioaccessibility and attenuating their inherent antioxidant and anti-inflammatory efficacy (5). Conventional off-flavor mitigation strategies, such as membrane filtration and chemical neutralization, often inadvertently strip away these bioactive compounds or introduce unwanted residues, thereby compromising product sustainability and quality (6). Consequently, there is a critical need for targeted, process-level bioconversion strategies capable of simultaneously remodeling the sensory profile and unlocking the functional potential of this underexplored matrix.

Microbial biotransformation is a promising approach for upgrading plant-derived by-products via microbial metabolism and extracellular enzymes. *Eurotium cristatum* has recently attracted attention in food fermentation due to its adaptability to fermentation-related stresses, including relatively elevated temperature and osmotic conditions (7, 8), as well as its ability to produce organic acids, aroma-active metabolites, and diverse extracellular enzymes (9). These metabolic outputs can improve flavor, mouthfeel, and overall quality of fermented products (10). Previous studies have reported that *E. cristatum* is commonly involved in traditional food fermentation, particularly as the dominant “golden flower” fungus in Fu brick tea. This long-standing fermentation background supports its use as a suitable microorganism for exploratory food-related biotransformation studies (11). Recent evidence further indicates that *E. cristatum* can express hydrolytic and oxidative enzyme activities during fermentation, thereby hydrolyzing glycosidically bound aroma precursors and phenolic conjugates and promoting oxidative conversion of phenolic substrates, which together reshape the volatile and phenolic profile of the fermentation system (9, 12, 13). As an aqueous distillation by-product, *L. chuanxiong* hydrosol mainly contains water-soluble constituents derived from the raw plant material. Phenolic compounds are commonly regarded as representative bioactive constituents in plant-derived aqueous matrices and have been associated with antioxidant, anti-inflammatory, and digestive enzyme inhibitory activities (14). In *L. chuanxiong*, phenolic acids, particularly ferulic acid and caffeic acid, have been reported as important constituents, and ferulic acid is often used as a quality-related marker (15). During *E. cristatum*-mediated fermentation, extracellular enzymes such as polyphenol oxidase may participate in the transformation of phenolic substrates through oxidation and degradation reactions. These transformations have been reported to be associated with reduced bitterness and astringency, as well as changes in sensory characteristics, in fermented tea and related plant-based matrices (10). Additionally, the fungus can synthesize bioactive secondary metabolites, such as *cristatumin A* and *D*, which have been reported to exhibit antioxidant and other functional properties (8, 16). These traits collectively position *E. cristatum* as a potential candidate for driving the biotransformation of challenging matrices such as hydrosols.

To overcome the aforementioned sensory defects and valorize this rich botanical resource, this study pioneered the application of *E. cristatum* in the submerged (liquid-state) fermentation of *L. chuanxiong* hydrosol. The temporal evolution of key aroma and taste determinants was systematically tracked using headspace solid-phase microextraction coupled with gas chromatography-mass spectrometry (HS-SPME-GC-MS), gas chromatography-olfactometry (GC-O), and electronic nose (E-nose) and electronic tongue (E-tongue). Concurrently, the biotransformed matrix was evaluated for its antioxidant capacity, anti-inflammatory activity, and inhibitory effects on selected metabolic enzymes to establish the relationship between chemical remodeling and functional outcomes. Collectively, this work aimed to clarify how *E. cristatum* fermentation reshapes the sensory profile and preliminary biofunctional properties of *L. chuanxiong* hydrosol. The findings provide a basis for the further valorization of essential-oil distillation side-streams as potential flavor-improving and biofunctional botanical ingredients.

## 2 Materials and methods

### 2.1 Materials

*L. chuanxiong* hydrosol was obtained from Yingu Aromatic Technology Co., Ltd. (Sichuan, China). *E. cristatum* (strain No. shbccd15081) was obtained from the Shanghai Collection Biotechnology Center. The cell lines RAW264.7 and HSF were purchased from Bolton Shanghai Biotechnology Co., Ltd (Shanghai, China).

MEB medium (BR), 2,2-diphenyl-1-picrylhydrazyl (DPPH), 2,2’-azinobis (3-ethylbenzothiazoline-6-sulfonic acid) (ABTS), α-amylase, α-glucosidase, lipopolysaccharide (LPS, Sigma-Aldrich), dexamethasone (Titan Scientific Co., Ltd., Shanghai, China), cytotoxicity detection kit (Beyotime), inflammatory cytokine ELISA kits for interleukin-6 (IL-6), interleukin-1 alpha (IL-1α), and tumor necrosis factor-alpha (TNF-α) (Beyotime), L-DOPA, arbutin, reactive oxygen species (ROS) detection kit (Beyotime), and superoxide dismutase (SOD) detection kit (Beyotime) were used in the study. Unless otherwise specified, all other chemicals were of analytical reagent grade and purchased from Sinopharm Chemical Reagent Co., Ltd. (Shanghai, China).

The aroma and taste profiles of the samples were analyzed using GC-MS (Agilent, United States), an E-nose (Super Nose, Herbin International Trading Co., Ltd., China), and an E-tongue (TS-5000Z, Insent, Japan). Additionally, an electronic analytical balance (Ohaus, United States), a constant-temperature incubator (Binder, Germany), a microplate reader (Thermo Fisher, United States), a high-speed centrifuge (Beckman Coulter, United States), and a microscope (Motic) were included.

### 2.2 Optimization of fermentation conditions

The single-factor experimental design method (11) was used to systematically investigate the effects of initial fermentation temperature, pH, inoculum size, shaker speed, fermentation time, and other process parameters on the fermentation performance of *E. cristatum*. Each factor was tested at five levels, with three parallel replicates per level. Optimal conditions were determined using the total phenolic content in the fermentation hydrosol as the evaluation index. A Box–Behnken design was then employed to identify significant factors influencing fermentation, which were further optimized using response surface methodology (RSM). Using the total phenolic content as the response variable, experimental design and data analysis were performed with Design-Expert software.

### 2.3 Electronic nose and tongue analysis

A volume of 2 mL each of the unfermented hydrosol, early-fermentation hydrosol (fermentation for 1–2 days), and late-fermentation hydrosol (fermentation for 6–7 days) of *L. chuanxiong* was accurately transferred into sealed headspace vials. The samples were equilibrated at 20°C for 50 min. Subsequently, headspace gas was automatically injected into the sensor array of the E-nose for detection and analysis (17). The operating parameters for the E-nose were as follows: flushing time, 80 s; measurement time, 100 s; pre-sampling time, 5 s; chamber flow rate, 450 mL/min; and initial injection flow rate, 300 mL/min (17). Detailed information regarding the sensor types, their target compounds, and sensitivity characteristics is provided in Table 1.

**TABLE 1:** Electronic nose sensor array and performance description.

Sensor	Compound type
S1	Aromatic compounds
S2	Nitrogen oxides, low molecular weight amines
S3	Sulfides
S4	Organic acid esters and terpenes
S5	Terpenes and esters
S6	Sterols and triterpenoids
S7	Oxygenated aliphatic compounds
S8	Amines
S9	Hydrogen compounds
S10	Furan compounds
S11	Volatile organic compounds (VOCs)
S12	Sulfides
S13	Ethylene
S14	Lactones and pyrazines

To assess alterations in the taste profile throughout the fermentation process, 10 mL aliquots of each sample—including unfermented hydrosol, early-fermentation hydrosol, and late-fermentation hydrosol of *L. chuanxiong* hydrosol—were diluted at a ratio of 1:5 with deionized water. Subsequently, the diluted samples were subjected to centrifugation at 5,000 rpm for 10 min to eliminate insoluble particulates (18). The clarified supernatants were then transferred into designated beakers for analysis using an E-tongue system. All measurements were conducted under controlled and standardized conditions, with sensor response data systematically recorded for subsequent evaluation.

A trained sensory panel consisting of 10 trained panelists (5 male and 5 female) was assembled to complement the instrumental analysis (18). All panelists underwent standardized training in taste assessment protocols and voluntarily participated with informed consent. All data were collected anonymously to protect participant privacy. Panelists were instructed to evaluate five key taste attributes—sweetness, sourness, bitterness, umami, and saltiness—for each sample. Sensory intensity for each attribute was scored on a 10-point scale (1 = extremely weak; 10 = extremely strong). Evaluation criteria and scoring reference standards are summarized in Table 2. The experimental protocol was in accordance with the Helsinki Declaration and ethical principles and was approved by the Ethics and Technology Safety Committee of Shanghai Institute of Technology (SIT-2026-LL32).

**TABLE 2:** Sensory evaluation criteria and scores.

Attribute (10 points)	Scoring criteria	Unfermented	Early fermentation (fermentation for 1–2 days)	Late-fermentation (fermentation for 6–7 days)
Sourness	1: Slightly sour			
	5: Noticeably sour			
	10: Sharply acidic and pungent			
Sweetness	1: Almost no sweetness			
	5: Moderate sweetness			
	10: Extremely sweet and cloying			
Bitterness	1: Slight bitterness			
	5: Moderate bitterness			
	10: Extremely bitter and unpleasant			
Umami	1: Almost no umami			
	5: Moderate umami			
	10: Very strong umami			
Saltiness	1: Almost no saltiness			
	5: Moderate saltiness			
	10: Extremely salty and astringent			

### 2.4 Analysis of characteristic substances

In this study, gas chromatography–mass spectrometry (GC-MS) was employed to analyze volatile compounds in the samples before and after fermentation.

The GC-MS system (7890A-5975C) was operated under the following temperature program (19): the initial oven temperature was set at 50°C and held for 2 min, then ramped to 120°C at 4°C/min and held for 3 min, followed by a further increase to 280°C at 8°C/min and held for 5 min. The injection temperature was set at 250°C. Nitrogen (N_2_) was used as the carrier gas at a flow rate of 1 mL/min, with a split ratio of 15:1. Mass spectrometry conditions were as follows: interface temperature, 280°C; ionization mode, electron impact (EI) at 70 eV; scan range, 33–550 amu; electron multiplier voltage, 1,000 V; and solvent delay time, 3 min.

Sample preparation: the fiber of the solid-phase microextraction (SPME) device was inserted into the gas chromatograph inlet and preconditioned at 250°C prior to sampling.

The same GC-MS conditions were employed, encompassing the chromatographic column, injection method, chromatographic parameters, column oven temperature program, and mass spectrometry detection settings. The transfer line temperature of the gas chromatography-olfactometry (GC-O) system was calibrated to 200°C, while the detector port temperature was maintained at 220°C (20).

The olfactometric panel comprised four trained assessors (three females and one male, aged 22–24), each with at least 6 months’ experience in fragrance recognition training (1 h per session, once per week). The panelists were asked to describe and rate the intensity of the odors on a 5-point scale (1 = very weak, 2 = weak, 3 = moderate, 4 = strong, 5 = extreme). An odor was deemed perceptible when at least two assessors identified it simultaneously. The intensity rating for each odor was derived from the average score provided by all panelists. The experimental protocol was in accordance with the Helsinki Declaration and ethical principles and was approved by the Ethics and Technology Safety Committee of Shanghai Institute of Technology (SIT-2026-LL32).

For semi-quantitative analysis, 5.0 mL of each sample was placed in a headspace vial, and 10 μL of p-xylene solution (0.15 mg/mL, 400 ppm) was added as the internal standard. After equilibration at 45°C for 20 min, the preconditioned SPME fiber was exposed to the headspace at 55°C for 30 min and then directly desorbed in the GC–MS injector. The relative content of each volatile compound was calculated from the ratio of its peak area to that of the internal standard. Odor activity values (OAVs) were calculated as OAV = C_*i*_/T_*i*_, where C_*i*_ is the concentration of the compound in water (μg/kg) and T_*i*_ is its odor threshold in water (μg/kg). Odor-threshold data were collected from published literature, and compounds with OAV > 1 were regarded as key aroma-active compounds.

For aroma-profile analysis, the odor descriptors obtained by GC–O were grouped into seven categories: floral, sweet, fruity, herbal, woody, green, and fatty. The overall intensity of each category was calculated by summing the arithmetic mean scores of the corresponding odor events. To validate aroma-profile changes at the sensory level, the same panel further evaluated the samples according to these seven predefined aroma attributes using the same intensity scale described above, and the intensity of each attribute was expressed as the average score of all assessors.

### 2.5 Cytotoxicity assessment

The cytotoxicity assessment was performed according to reported methods with appropriate modifications (21, 22). RAW264.7 and HSF cells were seeded in 96-well plates at a density of 5 × 10^3^–1 × 10^4^ cells/well with 100 μL of cell suspension, and incubated at 37°C with 5 % CO_2_ for 24 h. Cells were then treated with various concentrations of fermented *L. chuanxiong* hydrosol. For RAW264.7 cells, the concentrations of fermented *L. chuanxiong* hydrosol were 20, 10, 5, 1, 0.5, and 0.25%. For HSF cells, the concentrations of fermented *L. chuanxiong* hydrosol were 60, 30, 15, 7.25, 3.75, 1.88, and 0.94%. Different concentration gradients were used for RAW264.7 and HSF cells because the two cell lines exhibited different tolerance profiles toward the hydrosol samples. The dose ranges used in subsequent assays were selected on the basis of preliminary CCK-8 cytotoxicity screening to ensure that the tested concentrations remained within the non-cytotoxic range for each cell line.

After 6-96 h of incubation, CCK-8 reagent was added, and cells were incubated for an additional 1 h. Absorbance at 450 nm was measured using a microplate reader, and cell viability was calculated as follows:


$$\mathrm{Cell}\;\mathrm{viability}\left(\%\right)=\frac{O\,D_{\text{sample}}-O\,D_{\text{blank}}}{O\,D_{\text{control}}-O\,D_{\text{blank}}}\times 100$$

### 2.6 Antioxidant activity assays

DPPH Radical Scavenging Assay (23): Starting from the initial stage of fermentation, 1 mL of fermentation hydrosol was collected every other day, centrifuged at 5,000 rpm for 5 min, and the supernatant was obtained. The supernatant was diluted with distilled water to concentrations of 20, 40, 60, and 80%. Then, 3 mL of DPPH solution (0.02 mg/mL in ethanol) was added, and the mixture was allowed to react for 20 min. Absorbance was measured at 517 nm, and the scavenging rate (24) was calculated using the following equation:


$$\mathrm{DPPH}\;\mathrm{radical}\;\mathrm{scavenging}\;\mathrm{activity}\left(\%\right)=\frac{A_{\text{blank}}-A_{\text{sample}}}{A_{\text{blank}}}\times 100$$

ABTS Radical Scavenging Assay: The assay was performed based on the method of Erel et al. (25) with appropriate modifications. The fermented solution was mixed with the ABTS^+^ working solution and allowed to react for 30 min. The absorbance was measured at 734 nm, and the scavenging activity was calculated (26). Each sample was analyzed in triplicate.


$$\mathrm{ABTS}\;\mathrm{radical}\;\mathrm{scavenging}\;\mathrm{activity}\left(\%\right)=\frac{A_{\text{blank}}-A_{\text{sample}}}{A_{\text{blank}}}\times 100$$

Intracellular ROS Level Detection (27): HSF cells were seeded in 96-well plates at a density of 1 × 10^5^ cells/well and incubated at 37°C with 5% CO_2_ for 24 h. The experiment included a blank control group, an oxidative stress model group (induced with 200 μM H_2_O_2_), and treatment groups with different concentrations (3.75, 7.5, 15, 30%) of either stock solution or fermentation hydrosol. H_2_O_2_ (200 μM) was added simultaneously to the treatment groups. Each group included six replicate wells.

After 24 h of treatment, fluorescence intensity was measured using a microplate reader at an excitation wavelength of 488 nm and an emission wavelength of 525 nm, following the instructions of the ROS detection kit, followed by image capture using a confocal microscope. Antioxidant capacity was evaluated by comparing fluorescence intensities between groups (28).

SOD Activity Assay (29): After 24 h of treatment, the culture medium was removed and cells were washed three times with PBS. Cell lysate was then added in accordance with the SOD assay kit protocol, followed by lysis on ice for 30 min. The lysate was centrifuged at 12,000 rpm for 15 min, and the supernatant was collected. A colorimetric reaction was then performed according to the kit instructions, and absorbance was measured at 550 nm using a microplate reader to calculate SOD activity (30).

### 2.7 Anti-inflammatory activity assay

The anti-inflammatory activity assay was performed according to a reported method with appropriate modifications (31). RAW264.7 cells were seeded into 24-well plates at a density of 1 × 10*5* cells/well and incubated at 37°C in a humidified atmosphere containing 5 % CO_2_ for 24 h. The cells were then divided into the following groups: blank control group, model control group, positive control group, and sample treatment groups. In the blank control group, cells were cultured in complete medium only. In the model control group, cells were stimulated with lipopolysaccharide (LPS) at a final concentration of 1 μg/mL to induce an inflammatory response. In the positive control group, cells were treated with LPS (1 μg/mL) together with dexamethasone at a final concentration of 1 μmol. In the sample treatment groups, cells were treated with different concentrations of either unfermented hydrosol or fermented hydrosol together with LPS (1 μg/mL). Each group contained three replicate wells. After 24 h of treatment, the culture supernatants were collected, and the concentrations of inflammatory cytokines, including interleukin-6 (IL-6), interleukin-1 alpha (IL-1α), and tumor necrosis factor-alpha (TNF-α), were measured using ELISA kits according to the manufacturer’s instructions.

### 2.8 Hypoglycemic activity assays

α-Amylase Inhibition Assay (32): Different concentrations of fermentation hydrosol and 0.2 mL of α-amylase were added to the reaction system and incubated at 37°C for 10 min. Then, 1.0 mL of 3,5-dinitrosalicylic acid (DNS) reagent was added. After heating in a boiling water bath for 5 min, the absorbance was measured at 540 nm using a spectrophotometer to calculate the inhibition rate (33):


$$\alpha -\mathrm{Amylase}\;\mathrm{inhibition}\left(\%\right)=\frac{A_{\text{blank}}-A_{\text{sample}}}{A_{\text{blank}}}\times 100$$

α-Glucosidase Inhibition Assay (32): Fermentation hydrosol at various concentrations was mixed with 0.1 mL of α-glucosidase, followed by the addition of 0.1 mL of PNPG as substrate. The reaction was carried out at 37°C for 15 min. After the reaction, 0.5 mL of sodium carbonate solution was added to terminate the reaction. Absorbance at 405 nm was measured, and the inhibition rate was calculated (33):


$$\alpha -\mathrm{Glucosidase}\;\mathrm{inhibition}\left(\%\right)=\frac{A_{\text{blank}}-A_{\text{sample}}}{A_{\text{blank}}}\times 100$$

The IC_50_ values of the day-6 fermented hydrosol and the day-0 unfermented hydrosol were estimated by two-point linear interpolation between the two adjacent tested concentrations that bracketed 50% inhibition. For samples that did not reach 50% inhibition within the tested concentration range, IC_50_ values were reported as greater than the highest tested concentration.

### 2.9 Statistical analysis

Unless otherwise stated, all physicochemical and chemical assays were performed using three independent sample preparations, and the results are expressed as mean ± standard deviation (SD). Cellular assays were conducted with at least three independent experiments, and the number of technical replicate wells is specified in the corresponding method sections. Statistical analysis was performed using IBM SPSS Statistics 27.0. Differences among multiple groups were analyzed by one-way analysis of variance (ANOVA), followed by Tukey’s multiple comparison test. A value of *p* < 0.05 was considered statistically significant. Graphs were generated using GraphPad Prism (GraphPad Software, United States).

## 3 Results and discussion

### 3.1 Fermentation kinetics and process optimization

**FIGURE 1:**
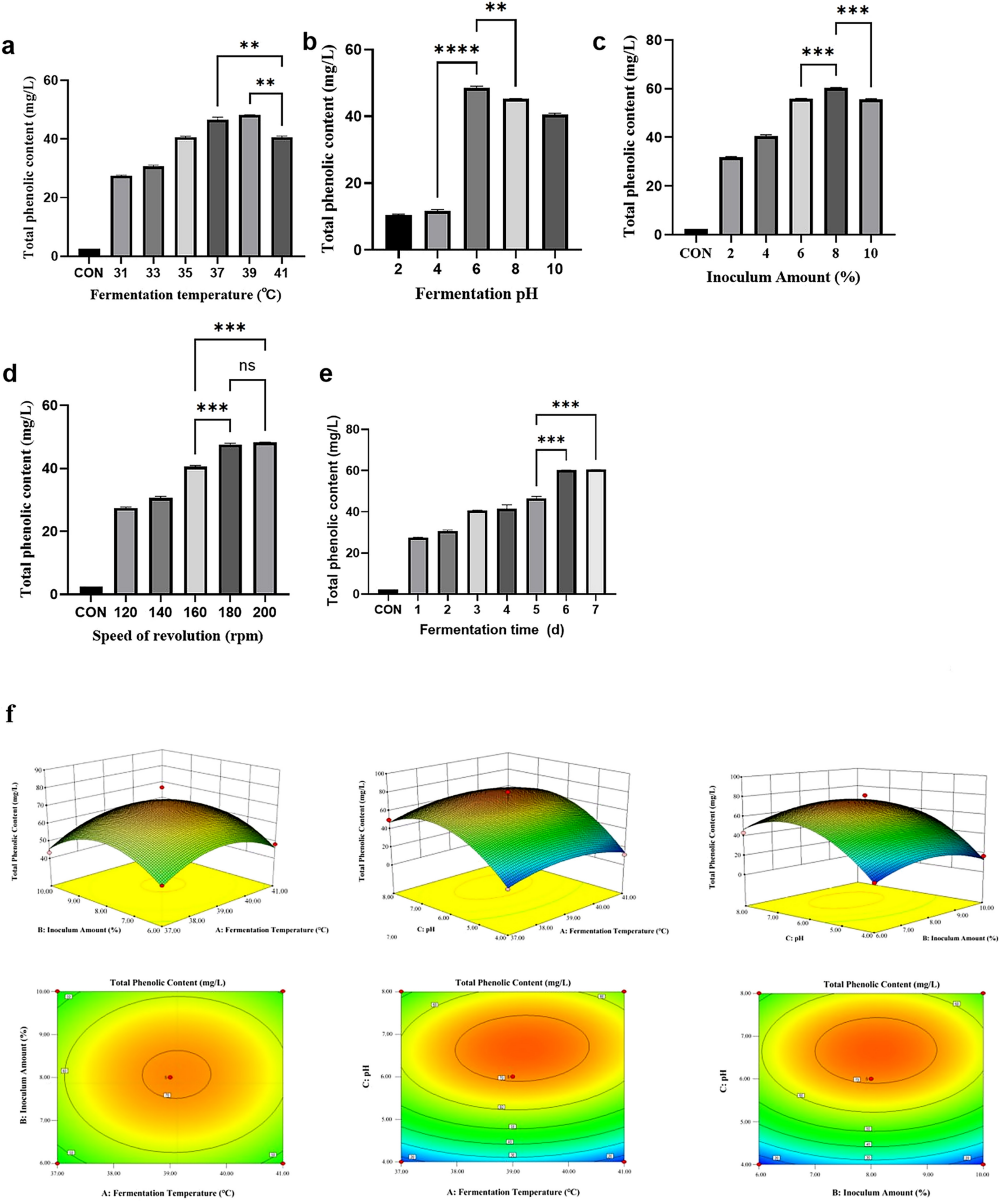
Effects of **(a)** fermentation temperature, **(b)** initial pH, **(c)** inoculum size, **(d)** shaking speed, and **(e)** fermentation time on the total phenolic content of *E. cristatum*-fermented *L. chuanxiong* hydrosol, and **(f)** response surface plots illustrating the interaction effects of fermentation variables on total phenolic content. CON, control. Statistical significance is indicated as follows: **P < 0.01, ***P < 0.001, ****P < 0.0001; ns, not significant.

Results from the single-factor experiments revealed the dynamic effects of key fermentation parameters on the release of bioactive constituents in *L. chuanxiong* hydrosol. As shown in Figure 1, increasing fermentation temperature, initial pH, inoculum size, stirring speed, and fermentation time led to a typical rise–fall pattern in total phenolic content (TPC). Specifically, TPC reached its maximum at 39°C, an initial pH of 6.0, and an inoculum size of 8%, with a stirring speed of 180 rpm and a fermentation duration of 7 days, and then decreased significantly with further increases in these variables (*p* < 0.05). This trend is consistent with the environmentally dependent metabolic activity of *E. cristatum* and its extracellular hydrolytic enzyme system. Under suitable temperature and pH conditions, enzyme activities such as β-glucosidase and esterase-like hydrolysis may facilitate the cleavage of glycosidic or ester linkages in bound phenolic conjugates, thereby promoting the release or conversion of phenolic compounds into more detectable forms. In contrast, excessively high or low environmental conditions may inhibit fungal growth or denature or inactivate key enzymes, ultimately impeding metabolite accumulation. Based on the fermentation conditions optimized through preliminary single-factor experiments and the sensory evaluation data, this study employed a Box–Behnken response surface design to conduct a multifactor optimization of interaction effects among the screened key variables. The single-factor experiments indicated that fermentation temperature, inoculum size, and pH each had a well-defined optimal range; therefore, these three variables were selected as the primary factors affecting fermentation performance.

Table 3 presents the analysis of variance (ANOVA) for the quadratic polynomial model developed using the Box–Behnken design (BBD) to assess goodness of fit. The model exhibited an *F*-value of 32.45 (*p* < 0.0001), indicating a highly significant regression. It confirmed that the selected fermentation temperature, inoculum size, and pH significantly affected the total phenolic content of the fermentation hydrosol, and that the second-order polynomial model constructed in this study satisfactorily fits the experimental data. In the significance test of individual factors, pH (C) had an extremely significant effect on total phenolic content (*p* < 0.0001) and was identified as the key factor influencing total phenolic accumulation during the fermentation of *L. chuanxiong* hydrosol by *E. cristatum*.

**TABLE 3:** Variance analysis of Box-Behnken experimental quadratic model.

Source	Sum of squares	df	Mean square	*F*-value	*P*-value	Significance
Model	7338.82	9	815.42	32.45	< 0.0001	Significant
A-Fermentation temperature°C	12.72	1	12.72	0.51	0.50	
B-Inoculum amount%	6.69	1	6.69	0.27	0.62	
C-pH	2548.88	1	2548.88	101.44	< 0.0001	
AB	1.19	1	1.19	0.047	0.834	
AC	15.62	1	15.62	0.62	0.46	
BC	2.09	1	2.09	0.083	0.782	
A^2^	527.33	1	527.33	20.99	0.0025	
B^2^	762.3	1	762.3	30.34	0.0009	
C^2^	3053.26	1	3053.26	121.51	< 0.0001	
Residual	175.89	7	25.13			
Lack of fit	55.35	3	18.45	0.61	0.6420	Not significant
Pure error	120.54	4	30.14			
Cor total	7514.71	16				
CV (%)	10.73					
*R* ^2^	0.9766					
*R* ^2^_*adj*_	0.9465					
Predicted *R*^2^	0.152					

The three-dimensional response surface plots in Figure 1f further visualize the combined effects of factor interactions on TPC. As shown, the response surfaces were pronouncedly convex, with densely distributed contour lines, indicating significant interactions among factors—particularly the pH-temperature interaction, which exerted the most prominent influence on TPC. Using Design-Expert software for numerical optimization, the theoretical maximum TPC (74.065 mg/L) was predicted at 39 °C, 7% inoculum, and pH 6.67. This prediction was validated experimentally, and the optimal conditions were determined to be 39 °C, a 7% inoculum, and pH 6.7, yielding a TPC of 71.25 mg/L, representing a significant improvement compared with the unfermented control.

### 3.2 Flavor improvement

**FIGURE 2:**
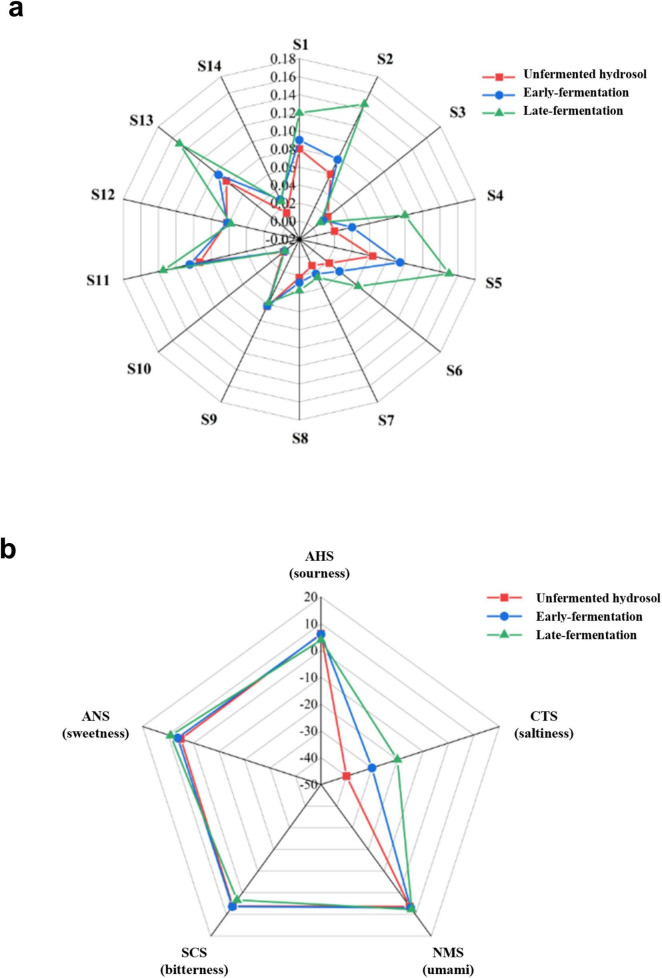
Radar plots of sensor responses from the electronic nose **(a)** and electronic tongue **(b)** for *L. chuanxiong* hydrosol samples at different fermentation stages: unfermented, early fermentation, and late-fermentation.

The sensor-array responses of the E-nose and E-tongue provide an intuitive reflection of the evolution of the overall flavor profile of *L. chuanxiong* hydrosol during fermentation. As shown in Figure 2a, the E-nose sensor array indicated that *E. cristatum* fermentation altered the volatile “fingerprint” of the hydrosol. Compared with the unfermented control and the early fermentation hydrosol, samples collected at the late-fermentation exhibited pronounced increases in sensors S4 (organic acid esters and terpenes) and S5 (terpenes and esters). Meanwhile, S11, which reflects the overall level of volatile organic compounds (VOCs), and S1 which reflects the overall level of aromatic compounds, also showed increasing trends, indicating that fermentation promoted the overall accumulation and intensification of aroma-related volatile constituents. These results provide rapid sensory evidence of the perceived improvement, characterized by diminished medicinal/pungent notes and a smoother aroma. In addition, sulfur-related sensors (e.g., S3/S12) and the furan-related sensor (S10) showed no substantial changes, implying that fermentation did not introduce a notable risk of sulfurous or furan-like off-odors. Overall, the E-nose results support that fermentation directionally optimized the volatile flavor spectrum of the hydrosol by weakening aromatic off-odor signals while enhancing terpene/ester signals and the total VOC signal.

Concurrently, the E-tongue radar plot (Figure 2b), together with quantitative sensory data (Supplementary Table S1), revealed targeted remodeling of taste attributes. Late-fermentation samples showed a significant decrease in bitterness (from 8.0 to 4.0), whereas sweetness (from 2.0 to 3.0) and umami-related indices increased. As noted by Guo et al. in their review of fermented tea beverages, microbial fermentation can significantly enhance the palatability of plant-based drinks by degrading bitter/astringent compounds (e.g., specific polyphenols or alkaloids) and generating sweet-tasting metabolites that mask their bitter and astringent properties (34). This sensory “debittering and sweetening” outcome essentially reflects the biotransformation of chemical constituents via fermentation.

### 3.3 Biotransformation of volatile compounds

In the unfermented hydrosol, 2,4-dimethylphenol was relatively abundant (4.06%). This phenolic compound is typically associated with strong medicinal and pungent smoky notes and is considered a major contributor to the poor sensory acceptability of *L. chuanxiong* hydrosol (35). GC–MS results indicated that it was completely undetectable (ND) in the post-fermentation samples (Supplementary Table S2). This observation suggests that oxidative enzyme systems secreted by *E. cristatum* (e.g., laccases and/or peroxidases) may cleave aromatic rings or promote the polymerization of volatile phenolics, thereby converting them into odorless and/or non-volatile products (9, 36). Previous studies have also shown that alkylphenols can be oxidized to phenoxy radicals, which then undergo oxidative coupling to form high-molecular-weight polymers or non-volatile oligomers (37). Such targeted biodegradation of off-flavor precursors explains the pronounced reduction in the “herbal/medicinal” attribute observed in the sensory evaluation (Supplementary Figure S1).

In contrast to the decrease in compounds with pungent odors, compounds with pleasant aromas and potential bioactivities increased after fermentation. Specifically, the relative abundance of α-terpineol increased from 0.04 % before fermentation to 6.10 % after fermentation, while D-limonene rose from 1.69 to 3.98% and citral increased from 1.02 to 5.09%. In addition, eugenol (4.07%) was produced during fermentation. Notably, these enriched compounds not only contribute elegant pine- and citrus-like notes, but have also been highlighted for notable anti-inflammatory and antimicrobial activities (38).

**FIGURE 3:**
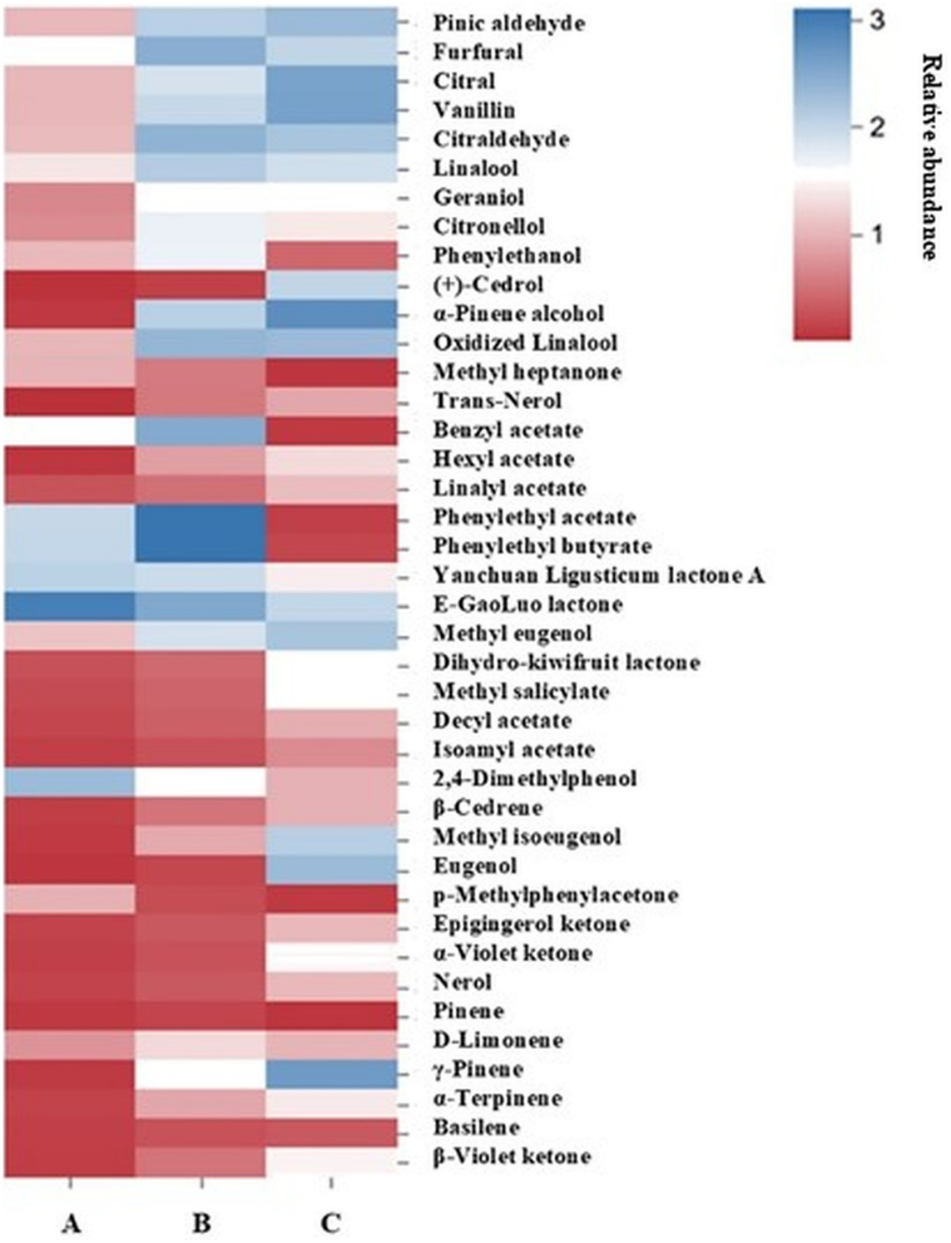
Heat map of representative differential volatile compounds identified in unfermented hydrosol **(A)**, early fermentation samples **(B)**, and late-fermentation samples **(C)**.

GC–O further elucidates the fermentation-driven, directional remodeling by comparing the relative abundance of key volatile markers in the unfermented hydrosol (A) and fermented samples (B/C). Based on reported odor-threshold data and the criterion of OAV > 1, 31 compounds were further screened as key aroma-active constituents (Supplementary Table S3). As shown in Figure 3 and Supplementary Figure S1, fermentation not only altered volatile abundance but also reshaped the relative sensory contribution of these odorants. Compared with the unfermented hydrosol, multiple pleasant aroma–associated oxygenated monoterpenes/terpene alcohols and their derivatives (e.g., α-terpineol, linalool/oxidized linalool, geraniol, citronellal, and (+)-cedrol) exhibited an overall upregulation in the fermented samples. In parallel, vanillin, which is closely linked to “sweet/vanilla-like” notes, was also enriched. In contrast, 2,4-dimethylphenol—an off-odor compound strongly associated with “medicinal/pungent smoky” perceptions in the unfermented hydrosol—was markedly reduced or attenuated during fermentation. Fermentation may decrease phenolic off-flavor precursors via transformations mediated by redox enzyme systems, consistent with the reduced aromatic-compound–related responses in the E-nose results and the sensory observation of diminished medicinal notes. Meanwhile, certain esters and lactones (e.g., ethyl hexanoate, ethyl isovalerate, ethyl decanoate, and several lactones/sandalwood lactones) showed stage-dependent increases during fermentation, suggesting esterification and/or biotransformation within the fermentation matrix. These compositional shifts corroborate the enhanced terpene/ester-related channels in the E-nose (e.g., S4/S5) and the macroscopic phenotypes from the E-tongue, namely reduced bitterness and improved palatability. Overall, the attenuation of phenolic off-odors, together with the enrichment of terpenes, sweet-aroma aldehydes, and selected esters, constitutes the chemical basis for flavor optimization following fermentation.

### 3.4 Hypoglycemic activity

**FIGURE 4:**
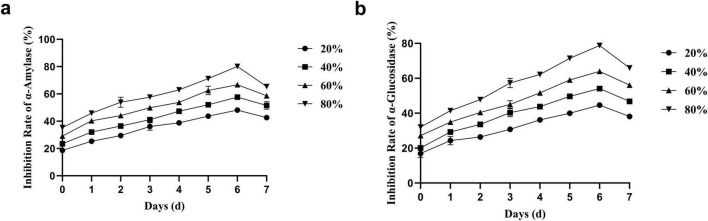
Inhibitory activities of hydrosol samples collected at different fermentation times against digestive enzymes. Day 0 was used as the unfermented reference: **(a)** α-Amylase inhibition; **(b)** α-Glucosidase inhibition.

Figure 4 presents the inhibition rates of α-amylase and α-glucosidase by the fermented hydrosol at different fermentation times and concentrations. For both enzymes, inhibitory activity was significantly dose-dependent (*p* < 0.05). On day 6 of fermentation, the 80% (v/v) fermented hydrosol achieved inhibition rates of 80% for α-amylase and 79% for α-glucosidase. In comparison, the unfermented hydrosol (0 day) at the same concentration showed inhibition rates of 35% for α-amylase and 32% for α-glucosidase. Therefore, fermentation markedly enhanced the inhibitory potential of *L. chuanxiong* hydrosol, corresponding to increases of 45% for α-amylase and 47% for α-glucosidase at the 80 % concentration. Based on the inhibition data shown in Table 4, the estimated IC_50_ values of the day-6 fermented hydrosol were 24.01% (v/v) for α-amylase and 31.32% (v/v) for α-glucosidase. In contrast, the IC_50_ values of the unfermented hydrosol were not reached within the tested concentration range ( > 80%, v/v).

**TABLE 4:** Inhibitory activities of day-6 fermented *L. chuanxiong* hydrosol at different concentrations.

Enzymes	% Inhibition				
	Hydrosol Concentration				IC_50_ (% v/v)
	20%	40%	60%	80%	
α-Amylase	48.09 ± 0.24	57.62 ± 0.39	66.70 ± 0.05	80.10 ± 0.30	24.01
α-Glucosidase	44.64 ± 0.18	54.11 ± 0.05	63.93 ± 0.46	78.76 ± 0.44	31.32

The increased enzyme inhibition may be associated with the enrichment of bioactive metabolites during fermentation. Guo et al. noted that fermentation not only increases total phenolic content but also converts macromolecular polysaccharides or bound phenolics into more bioactive small-molecule forms through microbial metabolism (34); these small molecules typically exhibit stronger affinity toward digestive enzymes.

### 3.5 *In vitro* antioxidant activity

**FIGURE 5:**
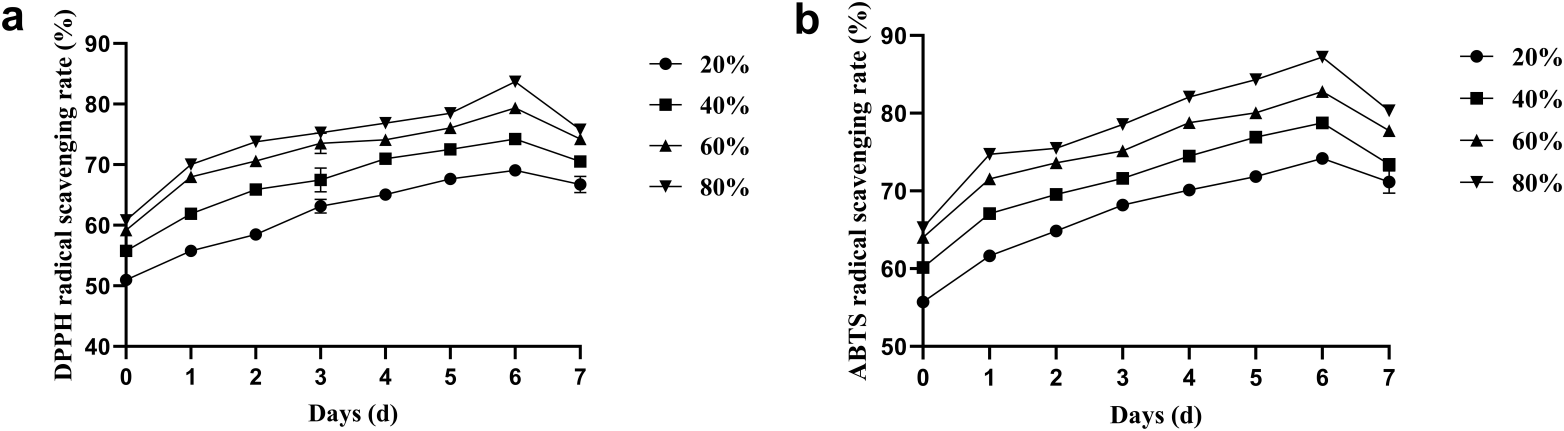
Antioxidant activities of hydrosol samples collected at different fermentation times. Day 0 was used as the unfermented reference: **(a)** DPPH radical scavenging activity; **(b)** ABTS radical scavenging activity.

The 2,2-diphenyl-1-picrylhydrazyl (DPPH) radical scavenging assay showed that the antioxidant capacity of the fermented hydrosol increased significantly with increasing concentration. As shown in Figure 5a, the DPPH scavenging activities of the fermented hydrosol exhibited a clear concentration-dependent trend across different fermentation times. The strongest scavenging activity was observed on day 6 of fermentation, when the 80% (v/v) fermented hydrosol reached 83%. The unfermented hydrosol (0 day) at the same concentration showed a DPPH scavenging rate of 61%. Thus, fermentation resulted in an increase in radical-scavenging capacity, corresponding to an increase of 22 at the 80% concentration.

Similarly, the ABTS assay confirmed that fermented *L. chuanxiong* hydrosol exhibited significantly more vigorous antioxidant activity than the unfermented hydrosol. As shown in Figure 5b, ABTS scavenging increased with sample concentration. Specifically, on day 6, the 80% (v/v) fermented hydrosol achieved an 84% ABTS scavenging rate. By comparison, the unfermented hydrosol (0 day) at the same concentration showed an ABTS scavenging rate of 65%, indicating an increase of 19% after fermentation. These results highlight the key role of fermentation in enhancing the radical-scavenging capacity of *L. chuanxiong* hydrosol in a concentration-dependent manner.

The improvement in antioxidant activity is closely associated with the increase in total phenolic content (TPC) in the fermentation system (Figure 1). It was reported that microbial enzymes secreted during fermentation (e.g., β-glucosidase) can hydrolyze phenolic glycosidic bonds and release free phenolic acids with strong reducing capacity (39). In the present study, GC–MS analysis (section 3.3) confirmed the accumulation of antioxidant-related compounds, including α-terpineol, vanillin, and coniferaldehyde, following fermentation. Gutiérrez-del-Río et al. suggested that these fermentation-derived terpenoids and phenolic small molecules can act as efficient electron or hydrogen donors to stabilize free radicals, thereby interrupting oxidative chain reactions (40). Therefore, the increased DPPH and ABTS radical-scavenging activities observed here may be associated with the fermentation-induced enrichment of phenolic- and terpene-derived compounds.

### 3.6 Intracellular antioxidant activity

**FIGURE 6:**
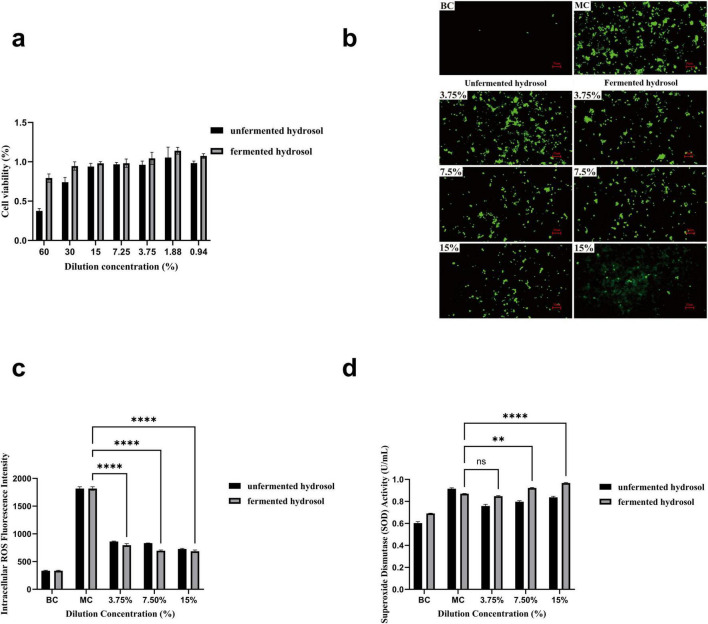
Effects of different concentrations of *L. chuanxiong* hydrosol and fermented hydrosol on oxidative stress in HSF cells: changes in cell viability **(a)**; representative fluorescence images of ROS detected by DCFH-DA staining **(b)**; intracellular ROS levels **(c)**; SOD activity **(d)**. BC, blank control; MC, the model group (H_2_O_2_ only). Statistical significance is indicated as follows: **P < 0.01, ****P < 0.0001; ns, not significant.

Prior to functional evaluations, a CCK-8 assay was performed to determine the safe concentration range for the samples. As shown in Figure 6a, the fermented hydrosol exhibited good biosafety toward HSF cells. At fermented-hydrosol concentrations up to 15%, HSF cell viability remained above 90%. To evaluate antioxidant efficacy in a cellular physiological context, an H_2_O_2_-induced oxidative stress model was established in HSF cells. Fluorescence microscopy images in Figure 6b show that the model group exhibited markedly increased green fluorescence intensity of the ROS probe, confirming successful induction of oxidative stress. In contrast, the fermented-hydrosol treatment groups displayed evident fluorescence quenching, which became more pronounced with increasing concentration. Quantitative analysis (Figure 6c) further confirmed that the ROS fluorescence intensities of the unfermented *L. chuanxiong* hydrosol at 3.75, 7.5, and 15% were 725, 674, and 587 RFU, respectively, indicating a relatively weak antioxidant effect. In comparison, the fermented hydrosol showed ROS fluorescence intensities of 653, 589, and 531 RFU at the same concentrations, respectively. These results indicate that the fermented hydrosol is not only effective *in vitro* (DPPH/ABTS), but can also exert intracellular ROS-scavenging effects after crossing the cell membrane.

The activity of the key intracellular antioxidant enzyme SOD was further determined. As shown in Figure 6d, the unfermented hydrosol had a limited effect on SOD activity, whereas the fermented hydrosol significantly enhanced SOD activity; notably, the 15% group reached 0.89 U/mg, exceeding that of the model group. The intracellular antioxidant results further supported the beneficial effect of fermentation-induced compositional remodeling. This effect may be related not only to the increased total phenolic content, but also to the enrichment of volatile constituents. First, GC–MS analysis confirmed that fermentation substantially increased the contents of coniferaldehyde and vanillin. Compared with macromolecular-bound polyphenols, these phenolic aldehydes possess higher lipophilicity and membrane permeability, enabling more efficient cellular entry and direct ROS scavenging (41). Second, α-terpineol and eugenol identified by GC–MS have been reported to activate the Nrf2 signaling pathway. Moratilla-Rivera et al. indicated that these small molecules can promote Nrf2 nuclear translocation and binding to antioxidant response elements (ARE), thereby upregulating endogenous antioxidant enzymes, including SOD (42). Therefore, the reduced ROS accumulation and increased SOD activity observed here indicate that fermentation-induced compositional changes may be associated with both intracellular ROS-scavenging responses and endogenous antioxidant-enzyme regulation.

### 3.7 Anti-inflammatory activity

**FIGURE 7:**
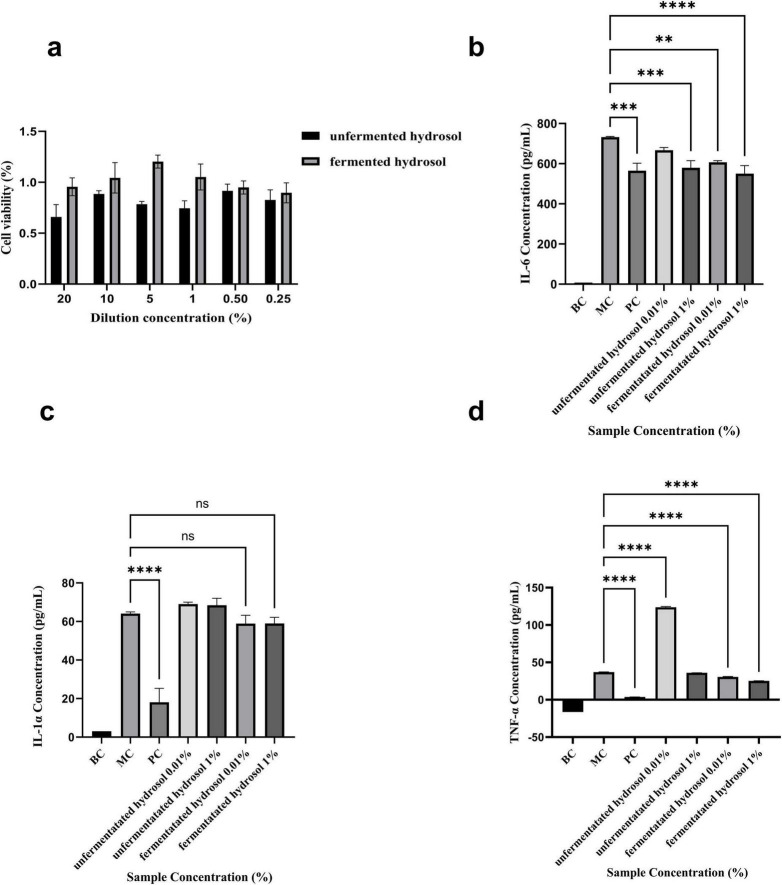
Effects of different concentrations of *L. chuanxiong* hydrosol and fermented hydrosol on inflammatory responses: changes in cell viability **(a)**; secretion of IL-6 **(b)**, IL-1α **(c)**, and TNF-α **(d)**. BC, blank control; MC, the model control group (LPS only); PC, positive control (LPS and dexamethasone). Statistical significance is indicated as follows: **P < 0.01, ***P < 0.001, ****P < 0.0001; ns, not significant.

Prior to functional evaluations, a CCK-8 assay was performed to determine the safe concentration range for the samples. As shown in Figure 7a, the fermented hydrosol exhibited good biosafety toward RAW264.7 cells. At fermented-hydrosol concentrations up to 20%, the RAW264.7 cell viability remained above 90%. Overexpression of inflammatory mediators is a key driver of skin injury and metabolic dysregulation. As shown in Figure 7, LPS stimulation markedly increased the secretion of pro-inflammatory mediators IL-6, IL-1α, and TNF-α in RAW264.7 macrophages compared with the blank control group (BC), indicating the successful establishment of the inflammatory model group (MC). However, treatment with 1% fermented hydrosol significantly suppressed the release of these cytokines; notably, its inhibitory effect on IL-6 was comparable to that of the positive control group (PC, dexamethasone) (*p* < 0.05) and was significantly stronger than that of the unfermented hydrosol at the same concentration. This anti-inflammatory potential may be associated with fermentation-induced changes in chemical composition. In addition to the increase in total phenolic content, GC–MS analysis (Supplementary Table S2) revealed significant enrichment of monoterpenes, such as α-terpineol and D-limonene, following fermentation. Salminen et al. reported that such terpene molecules can specifically block the phosphorylation cascade of the NF-κB signaling pathway, thereby suppressing transcriptional activation of downstream inflammatory genes (e.g., TNF-α and IL-6) (43). In addition, vanillin produced during fermentation has also been found to inhibit inflammasome activation (44). Together with previous reports on terpenoid- and vanillin-related inflammatory regulation, the decreased secretion of IL-6, IL-1α, and TNF-α suggests that NF-κB-related inflammatory signaling and inflammasome-associated responses may be involved in the anti-inflammatory effect of the fermented hydrosol.

## 4 Conclusion

**FIGURE 8:**
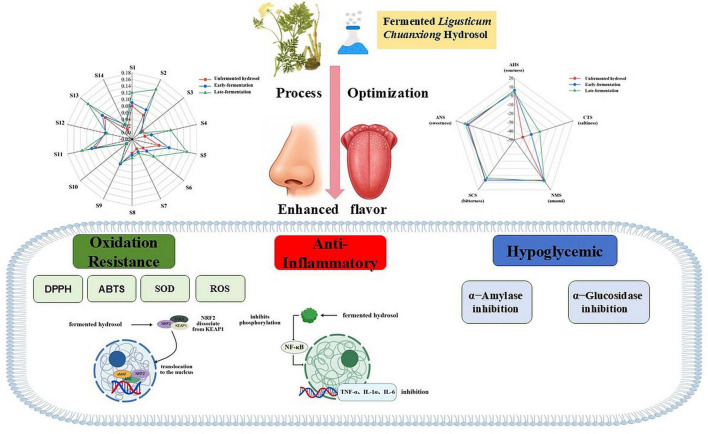
Schematic illustration of flavor and bioactivity enhancement in *L. chuanxiong* hydrosol fermented by *E. cristatum*.

This study suggests that biotransformation of *L. chuanxiong* hydrosol using *E. cristatum* is a feasible and eco-friendly strategy for valorizing this by-product from essential-oil distillation (Figure 8). The optimized fermentation process (39°C, pH 6.7, 7% inoculum) markedly improved sensory quality, as evidenced by the attenuation of off-flavor compounds (e.g., 2,4-dimethylphenol) and the enrichment of pleasant aroma-active metabolites (e.g., α-terpineol and D-limonene), while also enhancing multiple *in vitro* bioactivities, including radical-scavenging activity, digestive enzyme inhibition, anti-inflammatory potential, and protection against oxidative stress. It should be noted that the volatile-compound data obtained by GC-MS were based on an internal-standard-assisted semi-quantitative strategy and therefore mainly reflect relative changes among samples rather than absolute concentrations of all detected compounds. In addition, the observed bioactivity changes were discussed in association with fermentation-driven compositional remodeling, while the precise contribution of individual compounds and the related molecular pathways remain to be further clarified. Future studies will combine authentic-standard-based quantitative analysis, LC–MS/MS or HPLC-based characterization of non-volatile metabolites, compound-level activity verification, and molecular assays such as qPCR, Western blotting, immunofluorescence, and pathway-inhibition experiments to further validate key aroma-active compounds, active constituents, and possible antioxidant and anti-inflammatory pathways. Overall, these findings suggest that fermented *L. chuanxiong* hydrosol has promising application potential as a flavor-improving botanical additive and a candidate biofunctional ingredient for food-related or personal-care formulations. This work therefore provides a possible biotechnological approach for upcycling underutilized botanical co-products into value-added ingredients, facilitating the circular utilization of medicinal and edible plant resources.

## Data Availability

The original contributions presented in the study are included in the article/Supplementary material, further inquiries can be directed to the corresponding author.
