# Supplementary material for: Valorization of Ligusticum chuanxiong hydrosol via Eurotium cristatum biotransformation: mechanistic insights into flavor remodeling and functional enhancement
Source: Front Nutr. 2026 Jun 12;13:1852271. doi: 10.3389/fnut.2026.1852271 (PMC13307309; doi:10.3389/fnut.2026.1852271)
Supplement: Supplementary file 1 [file Data_Sheet_1.docx]

**Supplementary Material**

**Table S1** Sensory Evaluation Results of *Ligusticum chuanxiong* Hydrosol at Different Fermentation Stages

| Attribute (10 points) | Unfermented | Early-fermentation  (fermentation for 1–2 days) | Late-Fermentation  (fermentation for 6–7 days) |
| --- | --- | --- | --- |
| Sourness | 6 | 6 | 5 |
| Sweetness | 2 | 2 | 3 |
| Bitterness | 8 | 7 | 4 |
| Umami | 2 | 2 | 3 |
| Saltiness | 0 | 0 | 0 |
| Total Score (50 pts) | 18 | 17 | 15 |

**Table S2** Analysis of the relative contents of volatile compounds at different fermentation stages—unfermented (A), early-fermentation (B), and late-fermentation (C)—by gas chromatography–mass spectrometry (GC–MS).

| Category | CAS | Name | Relative content (%) | | |
| --- | --- | --- | --- | --- | --- |
|  |  |  | A | B | C |
| Aldehyde | 458-36-6 | Coniferaldehyde | 1.02 | 1.64 | 4.10 |
|  | 98-01-1 | Furfural | 2.05 | 2.06 | 3.10 |
|  | 5392-40-5 | Citral | 1.02 | 1.40 | 5.09 |
|  | 121-33-5 | Vanillin | 1.01 | 1.52 | 2.08 |
|  | 2385-77-5 | Citronellal | 1.06 | 2.07 | 3.69 |
| Alcohols | 000078-70-6 | Linalool | 1.56 | 1.69 | 2.80 |
|  | 000106-22-9 | Geraniol | 0.56 | 1.00 | 1.88 |
|  | 000106-24-1 | Citronellol | 0.61 | 1.21 | 1.62 |
|  | 000060-12-8 | Phenylethanol | 1.03 | 0.43 | 0.33 |
|  | 77-53-2 | (+)-Cedrol | 0.03 | 1.06 | 3.11 |
|  | 10482-56-1 | α-Terpineol | 0.04 | 1.64 | 6.10 |
|  | 1365-19-1 | Linalool oxide | 1.01 | 2.01 | 4.04 |
|  | 7212-44-4 | Nerolidol | ND | ND | 1.02 |
|  | 040716-66-3 | (E)-Nerolidol | ND | ND | 1.73 |
|  | 000106-30-9 | Ethyl hexanoate | 0.04 | 0.47 | 1.45 |
|  | 000535-77-3 | Benzylbenzoate | 1.63 | 1.57 | 1.48 |
|  | 000123-68-2 | Butyl acrylate | 6.07 | 4.10 | ND |
| Esters | 000103-45-7 | Phenethylacetate | 3.02 | 1.03 | ND |
|  | 62006-39-7 | Senkyunolide A | 9.36 | 6.36 | 5.35 |
|  | 81944-08-3 | E-Ligustilide | 7.02 | 6.02 | 3.05 |
|  | 004265-25-2 | Phenethyl butyrate | 3.04 | 2.06 | ND |
|  | 000109-21-7 | Ethyl benzoate | 2.07 | 1.09 | ND |
|  | 000115-95-7 | Linalyl acetate | ND | ND | 1.10 |
|  | 17092-92-1 | Dihydroactinidiolide | ND | ND | 2.02 |
|  | 119-36-8 | Methyl salicylate | ND | ND | 2.03 |
|  | 110-38-3 | Ethyl decanoate | ND | ND | 0.94 |
|  | 000084-66-2 | Diethyl phthalate | ND | ND | 0.53 |
|  | 108-64-5 | Ethyl isovalerate | ND | ND | 0.61 |
| Phenolics | 105-67-9 | 2,4-Dimethylphenol | 4.06 | 0.013 | ND |
|  | 000098-55-5 | p-Tert-butylphenol | 1.81 | 0.91 | 0.57 |
|  | 000093-15-2 | Methyl eugenol | 3.69 | 1.36 | 1.16 |
|  | 000097-53-0 | Eugenol | ND | ND | 4.07 |
|  | 000093-16-3 | Methyl-isoeugenol | ND | ND | 3.34 |
| Ketones | 000488-23-3 | p-Methylacetophenone | 0.24 | 0.13 | ND |
|  | 110-93-0 | Methyl heptenone | 0.03 | 0.01 | ND |
|  | 821-55-6 | 2-Nonanone | 0.04 | 0.01 | ND |
|  | 041702-63-0 | Epigingerone | ND | ND | 1.03 |
|  | 127-41-3 | α-Ionone | ND | ND | 1.79 |
|  | 79-77-6 | β-Ionone | ND | ND | 1.78 |
|  | 000089-48-5 | Methyl isobutyl ketone | ND | ND | 1.15 |
| Terpenes | 000099-83-2 | Terpinene | 6.05 | 3.01 | ND |
|  | 005989-27-5 | D-Limonene | 1.69 | 1.79 | 3.98 |
|  | 000100-42-5 | Styrene | 5.55 | 3.59 | ND |
|  | 000098-83-9 | α-Methylstyrene | 3.72 | 2.42 | 0.13 |
|  | 000099-85-4 | γ-Terpinene | 1.06 | 1.36 | 3.22 |
|  | 002713-09-9 | α-Pinene | ND | ND | 1.65 |
|  | 13877-91-3 | Ocimene | ND | ND | 1.23 |
|  | 28634-89-1 | β-Thujene | ND | ND | 1.71 |

**Table S3** OAV and aroma properties of 31 key active compounds

| **No.** | **CAS** | **Key aroma-active compound** | **Odor threshold (mg/kg) *** | **OAV** | | | **Aroma descriptor** |
| --- | --- | --- | --- | --- | --- | --- | --- |
|  |  |  |  | **A** | **B** | **C** |  |
| 1 | 98-01-1 | Furfural | 0.77 | 1.64 | 2.38 | 3.03 | Roasted aroma |
| 2 | 5392-40-5 | Citral | 5 | 1.02 | 1.05 | 1.95 | Citrus aroma |
| 3 | 121-33-5 | Vanillin | 0.37 | 1.23 | 1.23 | 1.65 | Sweet aroma |
| 4 | 106-23-0 | Citronellal | 0.52 | 1.69 | 1.89 | 6.23 | Rose-like aroma |
| 5 | 78-70-6 | Linalool | 1.082 | 1.63 | 2.64 | 69.16 | Floral aroma |
| 6 | 106-24-1 | Geraniol | 0.003 | 269.07 | 364.57 | 1391.84 | Rose-like aroma |
| 7 | 106-22-9 | Citronellol | 0.21 | 3.24 | 6.47 | 9.30 | Lemon-like aroma |
| 8 | 60-12-8 | Phenethyl alcohol | 0.39 | 8.47 | 6.35 | 1.23 | Rose-like aroma |
| 9 | 77-53-2 | (+)-Cedrol | 0.005 | 9.696 | 19.694 | 57.05 | Woody aroma |
| 10 | 98-55-5 | α-Terpineol | 0.33 | 1.743 | 3.475 | 5.89 | Pine-like aroma |
| 11 | 1365-19-1 | Linalool oxide I | 0.1 | 1.751 | 2.106 | 7.79 | Woody-floral aroma |
| 12 | 40716-66-3 | trans-Nerolidol | 0.12 | - | - | 27.72 | Woody-fruity aroma |
| 13 | 115-95-7 | Linalyl acetate | 1 | - | - | 0.30 | Lavender-like aroma |
| 14 | 80651-76-9 | Senkyunolide A | 6 | 4.15 | 3.57 | 1.57 | Herbal aroma |
| 15 | 93-15-2 | Methyleugenol | 0.0002 | 886.85 | 266.31 | 360.45 | Spicy aroma |
| 16 | 17092-92-1 | Dihydroactinidiolide | 0.000005 | - | - | 16714.1 | Musky-fruity aroma |
| 17 | 119-36-8 | Methyl salicylate | 0.04 | - | - | 2.112 | Herbal aroma |
| 18 | 110-38-3 | Ethyl decanoate | 0.82 | - | - | 0.131 | Fruity aroma |
| 19 | 108-64-5 | Ethyl isovalerate | 0.003 | - | - | 0.011 | Apple-like fruity aroma |
| 20 | 105-67-9 | 2,4-Dimethylphenol | 0.001 | 1181.03 | 3.454 | - | Smoky medicinal aroma |
| 21 | 6379-72-2 | Methyl isoeugenol | 10 | - | - | 0.011 | Sweet clove-like aroma |
| 22 | 97-53-0 | Eugenol | 0.06 | - | - | 410.88 | Clove-like aroma |
| 23 | 122-00-9 | 4-Methylacetophenone | 0.00015 | - | - | 1.091 | Cherry-like fruity aroma |
| 24 | 127-41-3 | α-Ionone | 0.0004 | - | - | 1266.31 | Violet-like woody powdery aroma |
| 25 | 40716-66-3 | Nerolidol | 0.68 | - | - | 36714.1 | Woody-fruity aroma |
| 26 | 99-86-5 | Terpinolene | 2.35 | 2.54 | 1.436 | - | Pine-like aroma |
| 27 | 5989-27-5 | D-Limonene | 0.034 | 34.73 | 76.28 | 122.36 | Citrus peel aroma |
| 28 | 99-85-4 | γ-Terpinene | 1.4 | 0.25 | 1.464 | 4.641 | Woody-citrus aroma |
| 29 | 80-56-8 | α-Pinene | 0.014 | - | - | 7.752 | Turpentine-like resinous aroma |
| 30 | 13877-91-3 | Ocimene | 0.034 | - | - | 19.90 | Herbal-floral aroma |
| 31 | 14901-07-6 | β-Ionone | 0.00008 | - | - | 5371.44 | Fruity aroma |
| Note: “–” indicates not detected.  Specifically, the odor-threshold values in water were referenced from *Compilations of Odour Threshold Values in Air, Water and Other Media* (van Gemert, 2011), unless otherwise indicated. | | | | | | | |


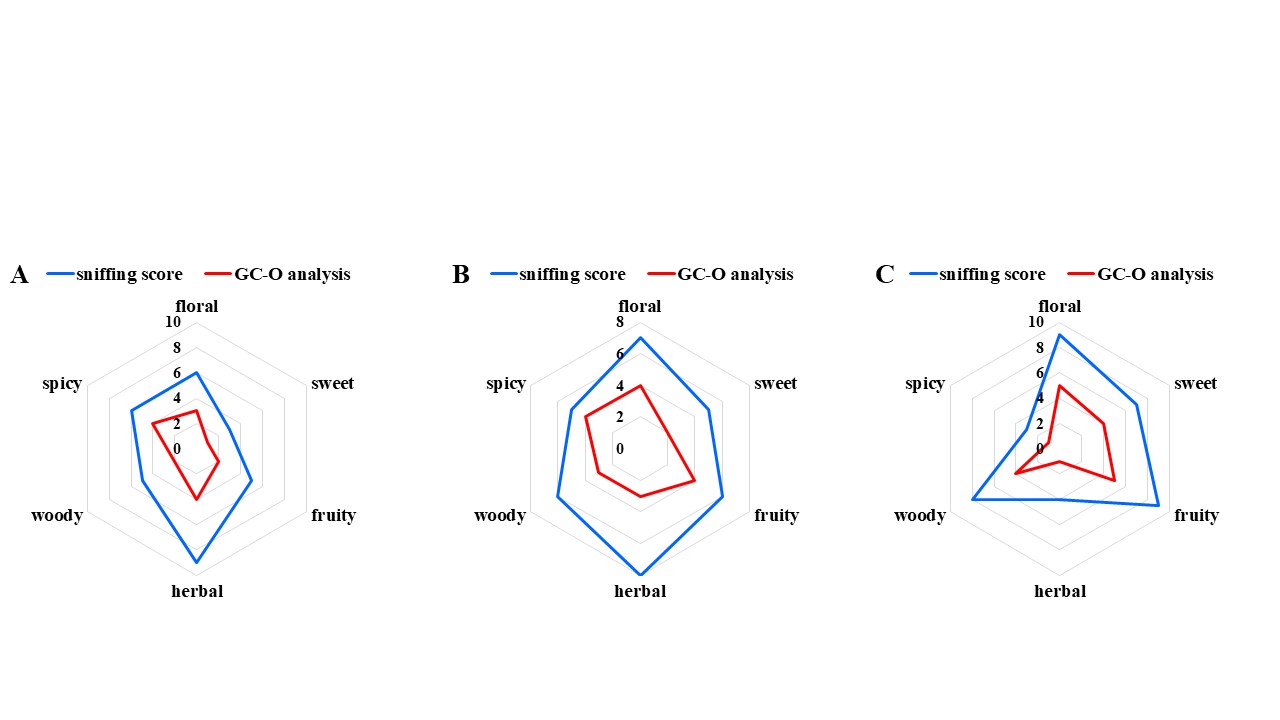


**Fig. S1**. Radar map of characteristic aroma profiles of hydrosol (A), early-fermentation (B), and late-fermentation (C)
